# Efficacy, model of delivery, intensity and targets of pragmatic interventions for children with developmental language disorder: A systematic review

**DOI:** 10.1111/1460-6984.12716

**Published:** 2022-04-20

**Authors:** Kristine M. Jensen de López, Jelena Kuvač Kraljević, Emilie L. Bang Struntze

**Affiliations:** ^1^ Clinic for Developmental Communication Disorders Institute of Communication and Psychology Aalborg University Aalborg Denmark; ^2^ Department of Speech and Language Pathology Faculty of Education and Rehabilitation Sciences University of Zagreb Zagreb Croatia

**Keywords:** developmental language disorder, intensity, intervention, model of delivery, pragmatic skills, targets

## Abstract

**Background:**

It is widely acknowledged that children with developmental language disorder (DLD) predominantly have difficulties in the areas of grammar and vocabulary, with preserved pragmatic skills. Consequently, few studies focus on the pragmatic skills of children with DLD, and there is a distinct lack of studies examining the effectiveness of pragmatic interventions.

**Aims:**

To carry out a systematic review of the literature on pragmatic interventions for children with DLD.

**Methods & Procedures:**

This systematic review was registered with PROSPERO (ID = CRD42017067239). A systematic search in seven databases yielded 1031 papers, of which 11 met our inclusion criteria. The included papers focused on interventions for children with DLD (mean = 3–18 years), enhancing oral language pragmatic skills, published between January 2006 and May 2020, and were based on a group‐study design such as randomized control trial or pre‐post‐testing. Study participants were monolingual speakers. The quality of papers was appraised using the Cochrane Risk of bias tool for randomized controlled trials.

**Outcomes & Results:**

There was a high degree of variability between the included intervention studies, especially regarding intensity, intervention targets and outcomes. The evidence suggested that pragmatic intervention is feasible for all models of delivery (individual, small and large group) and that interventions for pragmatic language are mostly focused on encouragement of conversation and narrative skills observed through parent–child interaction or shared book‐reading activities.

**Conclusions & Implications:**

This study highlights the importance of promoting and explicitly teaching pragmatic skills to children with DLD in structured interventions. A narrative synthesis of the included studies revealed that in addition to direct intervention, indirect intervention can also contribute to improving oral pragmatic skills of children with DLD.

**What this paper adds:**

**What is already known on the subject?**

- An increasing number of studies have shown that difficulties in acquiring pragmatic language is not only present in children with autism.

**What this study adds to existing knowledge?**

- Interventions for pragmatic language in children with DLD are mostly focused on encouragement of conversation and narrative skills, very often through parent–child interaction or shared book‐reading activities. Interventions that target language pragmatic are feasible for all models of delivery (individual, small and large group).

**What are the potential or actual clinical implications of this work?**

- The efficacy of the existing studies varies, and it is difficult to give recommendations regarding the intensity and duration of the specific intervention. In addition to offering pragmatic intervention directly from a specialist, pragmatic interventions can also be carried out indirectly if the intervention is under the continuous supervision of a specialist.

## INTRODUCTION

In addition to acquiring words and grammar during the early stages of language development children must acquire pragmatic rules that is, the knowledge necessary for the appropriate, effective, rule‐governed employment of speech in interpersonal situations (Ninio & Snow, 1996: 4). Pragmatic skills imply the use of language in different social situations and is traditionally studied in the fields of developmental psychology and linguistics. In psychology research focus has been on what Tomasello (2008: 7) called ‘the cooperative infrastructure of human communication’, that is, the cognitive abilities and social skills that lie beyond the use of language (Bates, 1976; Stephens & Matthews, 2014). Linguistic studies, on the other hand, consider pragmatic skills within the context of Austin's (1962) speech acts that relate to the speaker's intention behind their behaviour (Cameron‐Faulkner, 2014) as well as a scalar (quantity) implicature approach put forward by Grice (1975) (for more information on theoretical differences, see Falkum, 2019).

The development of pragmatics and the successful application of pragmatic skills are based on the interaction of two crucial skills: (1) adequate social skills, supported by social cognition; and (2) language skills through which the message is realised, by applying various pragmatic devices (Adams et al., 2005). According to Prutting and Kirchner (1987), pragmatic devices involve three aspects that are mastered synchronously: (1) verbal aspects that integrate elements such as variety of speech acts, topic selection, topic introduction, topic maintenance, turn‐taking initiation, turn‐taking response, revision, pause time‐overlap, cohesion, etc.; (2) paralinguistic aspects such as intelligibility, vocal intensity and quality, prosody, fluency; and (3) non‐verbal aspects such as physical proximity and contacts, body posture, foot/leg and hand/arm movements, gestures, facial expression and eye gaze.

This systematic review focuses on examining the use of the verbal and paralinguistic aspects in pragmatic interventions involving children with developmental language disorder (DLD).^1^

### Pragmatic intervention for children with DLD

DLD is a neurodevelopmental condition that emerges in early childhood and frequently persists into adulthood. Children with DLD have significant difficulty learning, understanding and using spoken language without any identifiable cause such as intelligence < 70, neurological damage, hearing impairment or other (Bishop et al., 2017). According to Norbury et al. (2016), at least 7.58% of 4‐ and 5‐year‐old children have language disorder as their primary need.

It is widely believed that children with DLD predominantly have difficulties acquiring vocabulary and grammar, while significantly less emphasis is placed on their difficulties in the field of pragmatics. For example, in a discussion of how knowledge about DLD (SLI) can contribute to theories of language development, our highly acknowledged international researcher in the field referred to the symptoms characteristic for DLD falling ‘safely outside the bounds of autism spectrum disorder’ (Leonard, 2014: 38). Moreover, recent studies conducted in the area of pragmatics indicate that children with DLD show difficulties in a number of areas including maxim of informativeness, that is, they show weak abilities in expressing and inferring information beyond what is explicitly said (Olsen et al., 2010; Katsos et al., 2011), applying pragmatic aspects of storytelling such as referencing, event content, mental state expressions and inferencing (Mäkinen et al., 2014) or inadequately answering questions (Befi‐Lopes et al., 2004). Moreover, a systematic review and meta‐analysis showed that children with DLD are also challenged within broader aspects of pragmatics and social cognition such as theory of mind (Nilsson & Jensen de López, 2016).

Defining effective interventions in pragmatics and in various clinical populations has been the goal of many studies. Unfortunately, only a few studies have focused on pragmatic skills of children with DLD. A systematic search of intervention studies for children with DLD within the domains of grammar, vocabulary, phonology, or pragmatics demonstrated significantly fewer intervention studies targeting pragmatics compared with the other domains (Murphy et al., 2016). In the initial stages of the current systematic review, we revealed dozens of systematic reviews on interventions that included pragmatic skills such as narrative (Adams et al., 2012) or conversation skills (Sng et al., 2018) but they mostly included populations of children with autism spectrum disorder (Parsons et al., 2017, Ke et al., 2017). In the Petersen (2010) systematic review of nine studies with mixed designs, he showed weak evidence that narrative intervention can improve some aspects of narrative macrostructure in children with language impairment. In another systematic review based on eight papers, the authors concluded, that children with language impairment and accompanying social communication/pragmatic deficits showed improvement in topic management skills (initiations, relevancy, topic maintenance, use of cohesion), narrative production, and repairs of inadequate or ambiguous comments after intervention (Gerber et al., 2012). However, in both systematic reviews, there was a high variability in the quality of the studies, a small number of participants who were primarily monolingual English‐speaking, small sample sizes, and a variety in methodological rigor, incomplete treatment protocols. These limitations prevented the authors from making empirically supported recommendations for changes in standard clinical practice. Therefore, further examination of the effectiveness of pragmatic interventions is necessary especially those that combine various pragmatic skills and analyse different intervention forms.

Pragmatic skills as an outcome of the interventions offered to children with DLD were first included in the late 1970s (Gallagher, 1990). Since then and in accordance with evidence‐based practice, interventions targeting pragmatic skills of children with DLD have become increasingly functionally oriented and based on ecologically valid activities. Each intervention is driven by a series of activities that were planned in advance (Justice et al., 2007; Hart et al., 2014) and include the following components:

- *Targets* that stand for the aspect of the recipient's functioning that is selected for change within the intervention (Hart et al., 2014).
- *Model of delivery* refers to whether the intervention is delivered individually (speech language pathologist (SLP) and child only) or in small or large group of children with the same disorder. Lieberman and Michael (1986) emphasise that while individual therapy serves as a direct support for establishing and stabilising specific speech and language behaviours, group therapy is additionally directed towards the development of appropriate interpersonal and social skills. Furthermore, therapy can be direct, meaning that the SLP or other specialist bears full responsibility for delivering the training; or indirect, meaning that a parent, supervised by a specialist, in some aspect helps deliver the training (Boyle, 2009).
- *Intervention intensity* stands for the amount of the intervention provided and is a key ingredient in determining intervention effectiveness (Zeng et al., 2012). Warren et al. (2007) have defined accumulated intervention intensity as a multiplication of three quantitative components—dose, dose frequency and total intervention duration. In other words, when information about the session length, frequency at which each treatment session occurs and the total period for which a specified intervention is provided are available, then information about intervention intensity can be defined (Zeng et al., 2012; Frizelle et al., 2021; Segura‐Pujol & Briones‐Rojas, 2021).

### The current study

Systematic reviews are informative and critical for evidence‐based practice and are useful for gaining overview of which interventions exist, on whether they are effective, and for gathering information regarding underlying mechanisms behind the effectiveness of a given intervention. To this date no systematic reviews have been constrained to investigating interventions aimed to affect pragmatic language of children with DLD.

The current review is one of a pair of reviews completed with a similar methodology and focuses on group studies providing pre‐ and post‐intervention measures. The other paper forming this pair focuses on intervention studies carried out using single‐case (non‐group) study designs, and on the content of the intervention (Jensen de López et al., under review). The two reviews focus on interventions which have measured outcomes in the domains of pragmatic oral language.

In this paper we conducted a systematic review and narrative synthesis addressing the following research questions:

- What is the reported efficacy and level of internal validity of the respective interventions?
- What were the targets of pragmatic interventions, model of delivery and who was the interventionist?
- What is the range of cumulative intervention intensities reported in the included studies?

## METHOD

### Systematic review

This systematic review was registered with PROSPERO (ID = CRD42017067239; Jensen de López et al., 2017): and is one of a series of SRs completed as part of European COST Action 1406. The aim of the action was to improve the understanding of intervention and service delivery for children with DLD across Europe as well as some additional partner countries. Our methods adhere to PRISMA guidelines for systematic reviews (Moher et al., 2015) and we present a narrative synthesis of the results.

### Search strategy and selection criteria

Searches within the Cost Action series of SRs were conducted to identify empirical peer‐reviewed articles, in any language, that related to oral language interventions with children with DLD. Seven electronic databases were included (Web of Science (including MEDLINE, SSCI), MEDLINE (PubMed), ERIC, PsychINFO, Cochrane Library, Scopus, and LLBA, and the search was conducted in two stages) (see Jensen de López et al., under review; Frizelle et al., 2021; and Murphy et al., 2016, for details). The search strings are provided in Appendix A in the additional supporting information. To include children with DLD searches included previous terminologies used to refer to this group of children or to subgroups within the umbrella of DLD, such as specific language impairment or primary language impairment. The specific search terms were discussed with members of the COST Action working group 1 and with a research librarian by the subgroup responsible for the global search (see Frizelle et al., 2021, for details).

#### Inclusion/exclusion criteria

Included papers met the following criteria:

- Research design: (1) randomized controlled trials (RCTs); (2) quasi‐experimental designs (non‐random assignment) with an element of control; and (3) cohort analytic designs, observational studies in which groups were assembled according to whether they have received the intervention, with control.
- Peer‐reviewed publication, published between January 2006 and May 2020.
- Participants with a mean age ≥ 3 and ≤ 18 years.
- Participants identified as having (1) DLD or an equivalent term such as primary language impairment, specific language impairment or developmental language impairment; and (2) difficulties on at least one oral language assessment (vocabulary, morpho‐syntax or discourse) falling below 1 SD below the mean. Studies were language impairment appeared secondary to those conditions identified by CATALISE criteria as precluding a DLD diagnosis (e.g., autism spectrum condition, learning disability) were not included. Only studies of monolingual children were included.
- Examined an oral language intervention which measured outcomes in the domain of oral pragmatics.

### Selection of papers and reliability of search procedures

**FIGURE 1:**
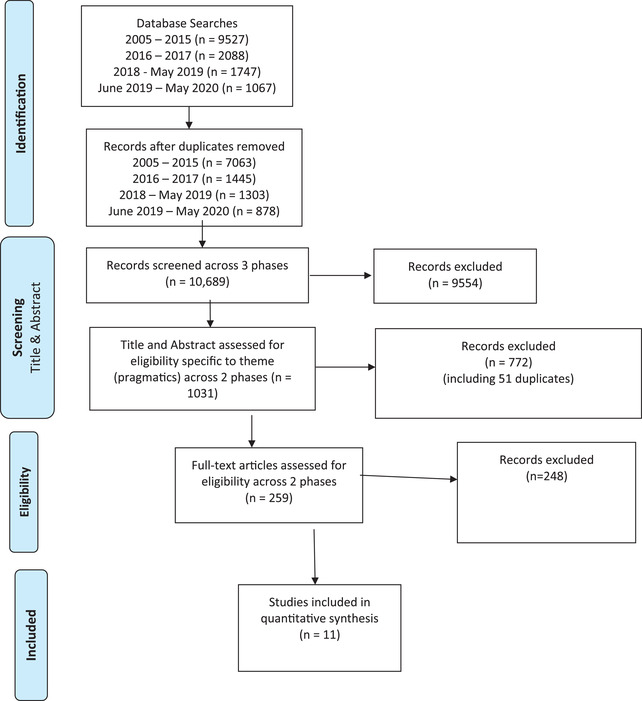
PRISMA flowchart showing the literature search process [Colour figure can be viewed at wileyonlinelibrary.com]

Selection of the papers was conducted on four consecutive steps using specialist software supporting systematic reviews (EPPI—Reviewer 4) and Excel:

- *Stage 1*: The initial search aimed to find papers that focused on different language domains (phonology, vocabulary, morphosyntax and pragmatics) and which would serve the WG1 subgroup within COST Action IS1406 for their further analysis given their specific interest. The papers were screened by researchers in the COST Action based on title and abstract analysing the previously prescribed criteria such as target group and intervention targets. A total of 20% of the papers were double screened by two independent reviewers (see Frizelle et al., 2021, for details).
- *Stage 2*: To identify papers specifically relevant to the domain of pragmatics two independent reviewers (K.J.L. and J.K.K.) screened all papers allocated to the domain of pragmatics on title and abstract. Due to the updated searchers this was carried out twice and yielded 1031 papers.
- *Stage 3*: The first stage of full text screening (*n* = 259) was completed against the inclusion/exclusion criteria by K.J.L. and J.K.K. Disagreements were resolved through discussion.
- *Stage 4*: Full text screening was completed by K.J.L., J.K.K. and E.B.S. on the papers that emerged from stage 3 and only those that focused specifically on oral pragmatic outcomes. Finally, 11 papers satisfied all initially prescribed criteria and were included in the analysis (Figure 1).

### Analytical approach

The included studies were examined in accordance with the Preferred Reporting Item for Systematic Reviews and Meta‐Analyses (PRISMA) framework. Furthermore, a range of categories consistent with the research questions were examined and coded. Narrative analyses of the aspects included in the research questions were then applied and provided in the results.

### Data extraction

K.J.L., J.K.K. and E.B.S. independently extracted the following data and tabulated it in an Excel spreadsheet or coded it in EPPI for the following categories; study design (RCT, quasi‐experimental, cohort analytical); participant variables (number, mean age, gender); treatment detail (intervention setting, treatment targets, model of delivery, unit of allocation, agent of delivery, intervention intensity (number and length of therapy session, dose frequency and total intervention duration); and pragmatic outcome measures and the findings).

### Risk of bias

**FIGURE 2:**
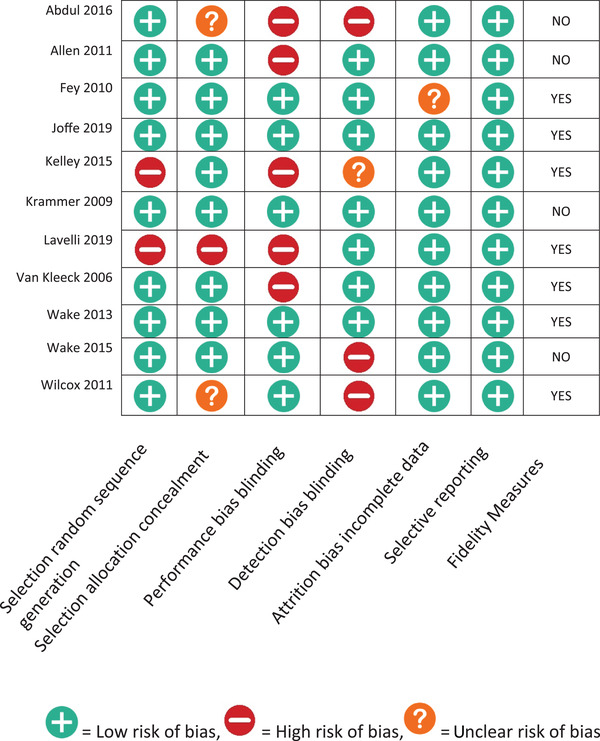
Critical appraisals of included studies [Colour figure can be viewed at wileyonlinelibrary.com]

K.J.L., J.K.K. and E.B.S. appraised the quality of each paper using the Cochrane Risk of bias tool for RCTs (Higgins et al., 2011). In total five different types of biases were evaluated: (1) selection bias that covers two aspects: random sequence generation and allocation concealment; (2) performance bias that stands for blinding of participants and personnel; (3) detection bias which evaluate blinding of outcome assessment; (4) attrition bias that control incomplete outcome data; and (5) reporting bias which deals with selective reporting. In addition, we analysed the faithful and correct implementation of the key components during the intervention (fidelity measurements). Bias risk was rated as high, low, or unclear using the Cochrane risk‐of‐bias tool for RCTs (Higgins et al., 2011). Two reviewers rated each article independently and disagreements were resolved by consensus. The critical appraisal for each paper is shown in Figure 2.

## RESULTS

A total of 11 studies examined language pragmatic as an outcome variable and were included in our analyses. An overview of the designs, samples, outcome variables and results are presented in Table 1. Only one of the included studies was carried out in a non‐English‐speaking country (Italy), with the remaining studies being conducted in the respective countries; Australia (three studies), UK (two studies), Canada (one study) and the United States (four studies). Seven of the studies were randomized controlled studies and the remaining studies were pre‐post‐test intervention‐control group studies.

**TABLE 1:** Summary of the included studies, characteristics of the sample, pragmatic language constructs and findings

		Study and aim		Characteristics			Outcome measures and findings		
Review number and authors		Design	Aim of the study	Country and language of children	Number and gender	Age	Pragmatic language construct	Main findings	Limitations
1	Abdul Aziz et al. (2016)	Pre‐post‐test with waitlist control and TD control	Evaluate the effectiveness of the speech regulation (SR) programme, and examine whether SR speech training improved planning and problem‐solving in children with DLD	Australia; English	*n* = 167; intervention T^1^ and T^2^ *n* = 47; waitlist control; intervention T^2^ and T^3^ *n* = 40; typical developing control *n* = 80 (59 girls)	4;0–6;7	Speech regulation including social speech (child–examiner interaction), private speech and inaudible speech	DLD group produced sign. More SR utterances after intervention T^3^ (*p* = 0.001, *d* = –0.87) and sign. more than the TD control group (*p* = 0.025, *d* = –0.35)	Effects of the intervention were based on calculations of a composite speech regulation score including social speech, private speech and inaudible speech
2	Allen and Marschall (2011)	Randomized controlled trial (RCT)	Identify whether communication parameters of children with DLD are influenced by parent–child interaction therapy (PCIT)	UK; English	*n* = 16; intervention *n* = 8; business as usual *n* = 8 (five girls)	8–9;06	Verbal initiations (spontaneous utterances with communicative intent, words or sounds with reference to shared activity or with clear intent to draw the adult's attention) Verbal responses (responses with utterances relevant to adult's previous utterance)	DLD group produced sign. More verbal initiations at follow‐up compared with control group (*p* = 0.001); no sign. Group differences on verbal responses, but ANOVA of interaction between time and group was sign (*p* = 0.05) with a large effect size (*n* ^2^ = 0.193)	
3	Fey et al. (2010)	RCT	To measure the effects of Fast‐For‐Word‐Language (FFW‐L) on the comprehension and production of narratives and complex grammar measured by a modified Narrative‐Based Language Intervention (NBLI) and language processing (phonological working memory) in children with DLD	USA; English	*n* = 23; FFW‐L/NBLI *n* = 7, NBLI/FFW‐L *n* = 7; waitlist/NBLI *n* = 9 (gender not stated)	6;9–8;43	Primary outcome measure: Narrative Language Ability Index (NLAI) from the (TNL)^b^; three oral narration subtests from the NLAI	No effect on oral language—very complex design	Completion rates for the FFW programme varied from 12% to 100%. Average for the FFW‐L/NBLI group was 53.57% and for the NBLI/FFW‐L group it was 46.43%)
4	Joffe et al. (2019)	RCT comparing four groups Waiting list control Narrative treatment Vocabulary treatment Combined treatment	Examine whether narrative, vocabulary or combined treatment improves narrative and vocabulary skills of children with LD	UK; English	*n* = 358; waitlist *n* = 85; narrative intervention *n* = 89; vocabulary intervention = 89; combined intervention *n* = 88 (132 = girls)	12;8	Two measures from the Expression, Reception and Recall of Narrative Instrument (ERRNI): recall and storytelling Narrative checklist (explicit narrative knowledge using Joffe 2011 ten‐item task) Story generation	*p*‐values are not reported for recall and storytelling. The narrative and combined narrative and vocabulary groups showed sign. Improvement compared with the waiting list control (*d* = 0.04, 0.06 and 0.34, 0.19); the narrative group showed sign. Improvement on the narrative checklist (*p* = 0.031, *d* = 1.50) compared with waiting control group; narrative and combined narrative and vocabulary groups showed sign. Improvements on story generation (*p* < 0.001, *d* = 0.95; *p* < 0.001, *d* = 0.54) compared with waiting control group	
5	Kelley et al. (2015)	RCT^a^	Efficacy study of Story Friends (an automated story book intervention for pre‐K curriculum) in preparation for a clinical trial allowing later refinement of implementation, measurement and to estimate effect sizes (children at risk)	n.s., but funded by the US Department of Education; English	*n* = 18; Story Friends intervention *n* = 9; business‐as‐usual *n* = 9 (11 girls)	4;0–4;11	Children's responses to literal and inferential comprehension questions related to stories based on the Assessment of Story Comprehension (ASC)	Intervention group produced sign. More correct answers to inferential questions compared with control group (*p* = 0.01, *d* = 1.10); group comparisons for answers to literal questions were n.s.	Small sample size
6	Kramer et al. (2009)	Pre‐post‐test between‐group design	Investigate whether dynamic assessment would differentiate children with normal language learning (NLL) from children with possible language learning difficulties (PLLD) in a narrative context	Canada; English	*n* = 17; PLLD, *n* = 5; NLL *n* = 12 (gender not stated)	n.s., Grade 3	Oral narratives across a range of components (e.g., quality of story, complexity of idea, vocabulary initiating event, etc.) A mixture of macro‐ and microstructure. Total score for DAI is reported	NLL group performed sign. Better on the DAI narrative scorings compared with the PLLD group	PLLD group is small
7	Lavelli et al. (2019)	Pre‐post‐test	Examine the impact of parent‐based story book reading on parental conversation strategy, engagement, conversation participation and linguistic production of children	Italy; Italian	*n* = 32; intervention *n* = 20; business‐as‐usual *n* = 12 (nine girls)	3;1–5;5	Conversation participation (number of communicative acts, initiatives and adequate answers)	n.s.	Small sample size
8	van Kleeck et al. (2006)	RCT	Examine the effects of a repeated one‐to‐one book‐sharing intervention on the literal and inferential language development of preschoolers with DLD	USA; English	*n* = 30; intervention *n* = 15; business‐as‐usual *n* = 15 (13 girls)	3;10–5;0	Preschool Language Assessment Instrument (PLAI) (Blank et al., 1978b) formal assessment of changes in literal language, as well as changes in inferential language skills	DLD group performed significantly better on PLAI (inference reasoning) compared with the control group	
9	Wake et al. (2013)	RCT (cohort)	Determine whether a population‐based intervention for children with DLD improves language and associated outcomes	Australia English	*n* = 200; intervention *n* = 99; business‐as‐usual *n* = 101 (68 girls)	5	Pragmatic skills reported by parents via CCC2 checklist	n.s.	
10	Wake et al. (2015)	RCT (cohort) 6‐year outcome of Wake et al. (2013)	Determine whether intervention for children with DLD at age 4 years would improve language and associated outcomes at age 6 years	Australia; English	*n* = 200; intervention *n* = 99; business‐as‐usual *n* = 101 (68 girls)	6	Pragmatic skills reported by parents via CCC2 checklist	Renfrew BUS Story: Information included in retelling and use of subordinate clauses	n.s.
11	Wilcox et al. (2011)	RCT	Evaluate the efficacy of the TELL (Teaching Early Literacy and Language) curriculum for children with DLD	USA; English	*n* = 118 (29 school classes); intervention *n* = 80 (19 school classes); business‐as‐usual *n* = 38 (10 school classes) (20% girls in TELL group and 34.2 girls in business‐as‐usual group)	3;9–5;3	Renfrew BUS Story: Information included in retelling and use of subordinate clauses	n.s.	

*Note*: ^a^The Kelley et al. study is a mixed design consisting of an RCT and an embedded repeated‐acquisition design. We exclusively report on data from the RCT part of the study.

^b^Test of Narrative Language (standardized instrument for evaluating the story comprehension, retell, and formulation skills of children 5–11 years of age).

The total number of children represented in the studies was 979, varying in sample size from 16 to 200 children (200 children were duplicates from Wake et al., 2013, presented in Wake et al.’s, 2015, follow‐up study so they were not included in the total number of children). The age of the children in the studies ranged from 3 to 12 years with just over half of the studies representing preschool children up to age 5 years (six studies).

RQ1: What is the reported efficacy and level of internal validity of the respective interventions?

Regarding the efficacy of the interventions, five studies (Abdul Aziz et al., 2016; Allen & Marshall, 2011; Joffe et al., 2019; Kelley et al., 2015; van Kleeck et al., 2006) showed significant effects in favour of the DLD group for the outcomes used to measure children's pragmatic language, with effect sizes ranging from medium 0.04 to large 1.50. The large effect size of *d* = 1.50 was produced using a 10‐item narrative checklist of explicit narrative knowledge by Joffe et al. (2019). Allen and Marshall (2011) measured two pragmatic language outcomes; verbal initiations and verbal responses; however, significant effects were only shown for verbal initiations. The areas of pragmatic language, for which the respective interventions proved to be effective, varied considerably from the child's ability to engage in speech regulation (social speech, private speech, and inaudible speech) to the ability to recall a narrative (ERRNI) and to respond adequately to inferential questions. The interventions that did not prove to be effective measured pragmatic language using the Narrative Language Ability Index (NLAI and Renfrew Bus Story), the CCC2 checklist and communicative acts (measured by number of verbal productions) and answers (measured as number of inadequate and adequate answers) as outcome measures. To summarize, the results gained across the included studies regarding the effects of the interventions did not show a clear picture of which aspects of pragmatics that certainly lead to improvement. Furthermore, several of the intervention studies held small sample sizes.

Regarding control of biases as the internal validity in the RCT studies, four studies did not report about performance bias and three studies indicated a risk of detection bias. Two papers showed high risk of selection bias whereby Lavelli et al. (2019) showed high bias for: random sequence generation and allocation concealment. There was an unclear risk of bias for two papers on selection allocation concealment and for one paper in detection bias and attrition bias. The high risk of bias and unclear bias in some studies reduced the reliability of the interpretation of the results.

A follow‐up period was conducted in two studies, but with a significant dropout of participants involved in the intervention (Fey et al., 2010; Joffe et al., 2019). Significant short‐term benefits were observed in two studies (Abdul Aziz et al., 2016; van Kleeck et al., 2006) however, the consolidation period was very short (8–12 weeks). Half of the included studies (five studies: Allen & Marshall, 2011; Kelley et al., 2015; Kramer et al., 2009; Lavelli et al., 2019; Wilcox et al., 2011) did not have a follow‐up period, meaning they did not assess the retention of knowledge and skills acquired during the intervention.

RQ2a: What were the targets of the pragmatic interventions?

We analysed the specific content of the included interventions to better understand what the main targets of the interventions were. In four papers, conversation was the target of the intervention, while narrative skills were targeted in the remaining seven studies. The conversation skills were based on verbal initiations, verbal responses, turn‐taking skill, question answering, etc. These skills have been confirmed in several previously published studies (Katsos et al., 2011; Befi‐Lopes & Rocha, 2004) as vulnerable areas that may be weak in children with DLD. In most interventions the SLP encouraged and observed the conversation between the child and his/her primary caretaker (usually the mother) or used shared book‐reading as the context of the intervention activity. Apart from Van Kleeck et al. (2006), which consisted of scripted book reading, all other studies were carried out within the context of a shared book‐reading activity (Joffe et al., 2019; Kelley et al. 2015; Kramer et al., 2009; Lavelli et al., 2019; Wake et al., 2013, 2015). In Van Kleeck et al. (2006), children participated in individual 15‐min shared book‐reading sessions twice per week with graduate and undergraduate research assistants from programmes in communication sciences and disorders. In some interventions, for example, that by Lavelli et al. (2019), parental responsiveness was combined with dialogic reading, that is, parents were first explicitly taught conversation strategies after which they applied them in the reading sessions with their child. Interventions that focused on narrative skills mostly measured various elements on the microstructure level (five papers: Fey et al., 2010; Kelley et al., 2015; Wake et al., 2013, 2015; Wilcox et al., 2011) and quite rarely elements on the macrostructure level, for example, Joffe et al. (2019) and Kramer et al. (2009).

Since one of the criteria for including studies was that the aim of the study was to change an aspect of language pragmatics, different aspects of dose form were sampled like private speech by Abdul Aziz et al. (2016) or verbal initiations and verbal responses by Allen and Marshall (2011). Interestingly, in Abdul Aziz et al. (2016), paralinguistic aspects (inaudible speech) that accompanied verbal aspects were observed and encouraged. It was operationally defined as any whispering, unintelligible muttering or silent lip movements that cannot be heard to be coded into one of the measured verbal categories. Representation of this type of pragmatic device in the included studies was however extremely low.

Furthermore, in some studies the trigger for improving pragmatic abilities such as narrative abilities were skills belonging to other language domains such as syntax by Wilcox et al. (2011) and vocabulary in by Joffe et al. (2019).

RQ2b: What model of delivery and who was the interventionist (agent) of the intervention delivery?

We analysed the model of delivery, unit of allocation, level of service delivery and setting of delivery in all 11 studies (Table 2).

**TABLE 2:** Included studies summarized by targeted pragmatic skill, as well as model, agent, dose and setting of intervention

Reference	Targeted pragmatic skill	Number of therapy session	Length of each session (min)	Dose frequency (per week)	Total intervention duration (weeks)	Model of delivery	Unit of allocation	Agent of delivery	Settings of delivery
Abdul Aziz (2016)	Conversation	10	30	Two times	5	Indirect	Group	Non‐specialist	School
Allen (2011)	Conversation	4	15	Once	4	Indirect	One to one	Non‐specialist	Clinic
Fey (2010)	Narrative	12	n.s.	Two or three times	5	Direct	Small group	Specialist	Clinic
Joffe (2019)	Narrative	18	45–60	Three times	6	Indirect	Small group	Non‐specialist	School
Kelley (2015)	Narrative	n.s.	n.s.	n.s.	14	Indirect	Small group	Non‐specialist	School
Kramer (2009)	Narrative	4	n.s.	Four times	1	Direct	One to one	Specialist	School
Lavelli (2019)	Conversation	32	n.s.	Four times	8	Indirect	One to one	Non‐specialist	Home
Van Kleeck (2006)	Conversation	16	15	Two times	8	Direct	One to one	Specialist	Preschool
Wake (2013)	Narrative	18	60	Once	18	Indirect	One to one	Non‐specialist	Home
Wake (2015)	Narrative	18	60	Once	18	Indirect	One to one	Non‐specialist	Home
Wilcox (2011)	Narrative	n.s.	150	Four times	n.s.	Direct	Large group	Non‐specialist	School

Interventions can either be direct, meaning that the SLP or a similar specialist bears full responsibility for delivering the training or indirect, meaning that a parent or other professionals, supervised by an SLP, in some respects help deliver the training (Boyle, 2009). In our included studies, four interventions were delivered directly and seven indirectly. It is important to emphasize that the type of model delivery in these studies had different goals. For example, in Joffe et al. (2019), based on an indirect model, the aim was to investigate the efficacy of teaching assistants in delivering narrative and vocabulary intervention to mainstream secondary school‐age students with language disorders. In studies based on a direct model of delivery, such as Fey et al. (2010), Kramer et al. (2009), Van Kleeck et al. (2006) and Wilcox et al. (2011), the aim was to examine the efficacy of a particular type of intervention in improving pragmatic skills. Direct intervention was always conducted by a specialist (SLP), while indirect intervention was either delivered by parents as in Allen and Marshall (2011), by a teaching assistant as in Joffe et al. (2019) or by some other trained professionals as in the intervention reported by Kelley et al. (2015) and Wake et al. (2013). In several of the interventions conducted indirectly children showed improvement, however, as emphasized by Allen and Marshall (2011), indirect invention relies heavily on parental motivation for intervention to succeed.

In six studies, the preferred unit of allocation was one to one, which is a common mode of SLP intervention. It was not only interventions based on direct mode that were organized on a one‐to‐one unit, but also some interventions that were based on indirect mode were conducted on an individual basis. That was the case in the studies by Allen and Marshall (2011), Lavelli et al. (2019) and Wake et al. (2013, 2015). Nearly half of all studies had a small group as their main unit of allocation (Abdul Aziz et al., 2016; Fey et al., 2010; Joffe et al., 2019; Kelley et al., 2015), whereas in Wilcox et al. (2011) a large group was the unit of allocation.

Five studies took place in mainstream schools: one in a pre‐school, two in the clinic and three in the child's home.

RQ3: What is the range of cumulative intervention intensities reported in the included studies?

To appreciate the role of intensity for the effects of the individual studies, we evaluated three quantitative aspects of dosage: one component of dose (the session length), dose frequency and total intervention duration (Table 2).

All these aspects were highly variable among the studies. The total number of intervention sessions ranged from four (e.g., Allen & Marshall, 2011; Kramer et al., 2009) to 32 (Lavelli et al., 2019). In two studies (Kelley et al., 2015; Wilcox et al., 2011) information about total number of intervention sessions was not stated, and it was not possible to calculate. Session length ranged from 15 min (Allen & Marshall, 2011; Van Kleeck et al., 2006) to 150 min (Wilcox et al., 2011). In Allen and Marshall (2011), parents were asked to play/carry out activities for 15 min, while in Wilcox et al. (2011) the primary aim was to examine the efficacy of a new preschool oral language and early literacy curriculum package called Teaching Early Literacy and Language (TELL). Since TELL was part of the curriculum, it was offered four times weekly with 150 min of instruction scheduled every day. In four studies (Fey et al., 2010; Kelley et al., 2015; Kramer et al., 2009; Lavelli et al., 2019) no information was reported about session length. Except for Lavelli et al. (2019), dose frequency and total intervention duration were the most consistently reported data about dosage across the analysed studies. Dose frequency ranged from once to four times per week. Wilcox et al. (2011) was the only study not to report information about total intervention duration. In the other studies, duration intensity ranged from 1 to 18 weeks. The shortest duration was reported by Kramer et al. (2009). This study was based on dynamic assessment intervention that is defined by a test–teach–retest format. All three children included in the study participated in two mediation sessions; the first mediation session took place on an average of 3 days after the test phase, and the second mediation session took place the day after the first session except in one case where the session took place 2 days later. In all cases, both mediation sessions were conducted within 1 week. The longest duration within the included studies was for the intervention conducted by Wake et al. (2013, 2015) and consisted of an 18‐session programme (three 6‐week blocks of weekly sessions, starting every 3 months) delivered by the authors on a one‐on‐one basis.

## DISCUSSION

The choice of effective service delivery is a major concern for practitioners, service commissioners and policymakers. Narrative synthesis of the results of studies within systematic reviews are widely regarded as one of the most valued and reliable ways of synthesizing research evidence (Marshall et al., 2011). To our knowledge, our systematic review is the first review to exclusively investigate interventions that target pragmatic language‐focused interventions in children with DLD including various dose forms. The purpose of this study was to systematically evaluate peer‐reviewed articles from the last 15 years concerning the effect and content of pragmatic based speech–language interventions for children with DLD. There are still mis‐conceptual believes that children with language disorders show exclusively difficulties with the lexicon and grammar, whereas pragmatic difficulties primarily are seen in children with autism spectrum disorder. However, several studies have pointed to the fact that difficulties within the pragmatic domain also are the hallmark of children with DLD (Befi‐Lopes & Rocha, 2004; Olsen et al., 2010; Katsos et al., 2011; Mäkinen et al., 2014). These results align with recent studies showing that children with DLD are delayed in their understanding of ToM (Nilsson & Jensen de López, 2016), which may be mediated by their delayed language.

Narrative synthesis of the 11 papers that met the inclusion criteria for this study showed a high degree of diversity in reporting about components relevant for confirming intervention effectiveness. That was especially the case with targets, intervention outcomes and intensity of the intervention. Moreover, the included studies were not uniform in terms of risk‐of‐bias reporting and this might affect the outcomes and conclusions of the studies (Higgins et al., 2011). In our study, performance and detection bias were the most frequent aspects with risks of biases that might influence the results. Despite this diversity among the included studies, some important conclusions can be drawn from the results of this systematic review.

First, when dealing with pragmatic language, researchers are mostly oriented towards conversation and narrative skills. A similar pattern appears for interventions based on single case designs (Jensen de López et al., under review). In relation to pragmatic devices, researchers are mostly focused on the verbal pragmatic aspects, and to a lesser extent on those that are paralinguistic. It means that verbal pragmatic elements such as speech acts, topic selection, turn‐taking, etc. are more often the focus of SLP research than paralinguistic elements like prosody and fluency, or metalinguistic skills like explicit awareness of pragmatic rules and when these have been violated.

What seemed a bit surprising in our results is that even for pragmatic interventions; the dominant unit of allocation is one‐to‐one which is a common mode of intervening in speech, language, and communication disorders. There are advantages of individual interventions such as the full attention of the SLP to the one client and his/her needs or better management and control of multiple goals and targets during the intervention, and less opportunities for distraction of the child. Group intervention was also present in some of the included studies but to a lesser extent. An important advantage of group intervention in addition to establishing specific speech and language behaviours is that it is directed towards the development of appropriate interpersonal and social skills (Lieberman & Michael, 1986) and is embedded in an ecological valid setting. These kinds of skills are important building blocks for the development of pragmatic language abilities and intrinsic aspects of pragmatic language (Adams et al., 2005). Yet few interventions seemed to have capitalised on these specific skills.

The included studies also differed in terms of reporting the intensity of the interventions. Dose frequency and total intervention duration are the most consistently reported data about intensity. In two recently published systematic reviews the authors dealt with papers in which dosage was statistically analysed (Frizelle et al., 2021; Segura‐Pujol & Briones‐Rojas, 2021). In Frizelle et al. (2021), the results showed that frequent interventions (two to three times per week) that target language goals for short periods or less frequent interventions (once per week or fortnight) targeting language goals for longer yield the best outcomes in relation to composite language measures. The preliminary conclusions of Segura‐Pujol and Briones‐Rojas (2021) suggested that greater stimuli variability provided better results in treating morphology, whereas greater exposure to stimuli does not necessarily translate into improved performance for vocabulary tasks. Similar conclusions cannot be drawn from data obtained in our pragmatic intervention review since we were not restricted to papers in which dosage was experimentally manipulated as our goal was to analyse the range of intensities in the interventions. In fact, the intervention with the longest intensity (Wake et al., 2013, 2015) did not show statistically significant changes.

Furthermore, there are differences in the effectiveness of the interventions among the included studies and some studies did not report effectiveness. Therefore, the results of the efficacy of the interventions included in our review did not show a clear picture of which interventions were most effective for improving pragmatic language. In addition, if we add the fact that the analysed studies had different research objectives—in some of them the aim was to test the intervention effectiveness, while in other studies intervention was the means for achieving a different goal — this further complicates the drawing of reliable conclusions.

There seems to be a clear need for further research that can identify pragmatic interventions that are effective for children with DLD and across languages other than English. It is also important that future intervention studies include outcome measures with high levels of ecological validity to certify that there has been a transfer of the intervention to the everyday activities of the child. Finally, it is equally of importance to establish what type of content and context, but also which level on intensity is most effective for treating pragmatic language disorders in children with DLD. As mentioned earlier there are a vast amount of pragmatic intervention studies for children with autism disorder, however due to the diversity of the two groups we cannot expect these existing interventions to be the most effective options for children with DLD.

### Implication for practitioners

The results of this review point in several directions in terms of their relevance for clinical work. Lack of pragmatic intervention studies for children with DLD calls on urgent reconsiderations of speech–language practice. There are an increasing number of studies that report on pragmatic difficulties in children with DLD across age groups, so encouraging different aspect of pragmatic must become an inevitable part of language intervention.

Although language skills intertwine and develop in tandem, it cannot always be expected that a child who has been exclusively taught vocabulary and grammar during the intervention will show progress in pragmatic skills. However, what should be kept in mind is the mandatory inclusion of pragmatics in intervention programmes for children with DLD. It is also important to be focused on all elements of conversational skills (introduction, reintroduction and maintenance of the topic or turn‐taking skill, etc.) as well as on both levels of narrative abilities; those related to linguistic knowledge (microstructure level) and those related to story structuring (macrostructure level). In this sense, all available interventions should be described in detail for SLPs to deliver them easily and consistently.

The results of our review point to the contribution of indirect intervention as an effective mode. This service delivery model is especially important in a situation where there are not enough SLPs or when there are long waiting lists. In that case, two facts should be kept in mind: (1) as stated by Allen and Marshall (2011), the motivation of parents to participate in the intervention is an important variable: even the shortest intervention requires involvement and consistency that sometimes parents cannot ensure, and this may influence on the effectiveness of the intervention; and (2) involvement of a third person in the implementation of intervention does not mean replacement of the SPL. Parents can be good partners if they are motivated and well mentored by a speech–language therapist or specialists in the field, and they also have good knowledge of which areas of pragmatics are challenging for their child (Jensen de López et al., 2021) but this does not mean that they can completely replace a speech–language therapist. As Fey et al. (1993) stated, treatment delivered by SLPs is more consistently effective because a well‐trained clinician can use procedures of a uniformly high quality even if the children differ considerably in their developmental status. On the other hand, the notion of individual differences also stands for parents providing intervention to their child. It is the case for some parents that they find it extremely difficult to learn treatment procedures and to implement them with their children regardless of the quality or intensity of parent training (Fey et al., 1993).

Our results showed that pragmatic language interventions were appropriate for group therapy. Besides verbal aspects of pragmatic skills, group intervention can directly influence non‐verbal aspects such as the child's social interaction skills within which pragmatic skills are realised. In doing so, several other contextual factors that affect the success of group intervention should be considered, such as the size and homogeneity of the group, group dynamics and control of the involvement of all members in therapeutic activities (Matić et al., 2018).

### Limitation

This systematic review has some limitations that need to be addressed. First, there were concerns with the methodological quality, small sample sizes, and the variability in the duration of the interventions. In addition, there is a lack of research reported on pragmatic interventions with non‐English speaking children with DLD. Second, due to the diversity of targets and outcome measures, it was impossible to compare these directly across the included studies. Third, weak control of selection bias which is a relevant component for interpreting studies inevitably affected our conclusion about intervention effectiveness. In addition to this, half of the studies did not report about follow‐up measures which is relevant to provide evidence about the sustainability of the effect. All these limitations do not detract from the need for further work to develop effective pragmatic interventions for children with DLD. Rather, they point to the fact that researchers and practitioners must engage in developing standard outcome measures to capture pragmatic language if we are ultimately to create reliable interventions for children with DLD.

## CONCLUSIONS

Several systematic reviews focusing on pragmatic interventions are available today, but none of them include children with DLDs. Therefore, the motivation of this research was to fill the gap in the literature and to give directions for clinical work.

Despite a high diversity in the 11 included studies regarding intervention targets, intensity and outcomes, several relevant conclusions, especially for clinicians, can be drawn. The evidence suggested that interventions for pragmatic language are mostly focused on encouragement of conversation and narrative skills, very often through parent–child interaction or shared book‐reading activities. Further, they are feasible for all models of delivery (individual, small and large group). Pragmatic interventions can also be carried out indirectly if the motivation of non‐specialists to participate in the intervention is ensured and if their training for the implementation of therapy is under the continuous supervision of a specialist.

## CONFLICTS OF INTEREST

The authors have no known conflict of interest to disclose.

## Supporting information

Supporting Information

## NOTE

1 DLD has previously been referred to as specific language impairment (SLI).
