# Supplementary material for: Efficacy, model of delivery, intensity and targets of pragmatic interventions for children with developmental language disorder: A systematic review
Source: Int J Lang Commun Disord. 2022 Apr 20;57(4):764–81. doi: 10.1111/1460-6984.12716 (PMC9544814; doi:10.1111/1460-6984.12716)
Supplement: Supplementary file 1 — Supporting Information [file JLCD-57-764-s001.docx]

**Appendix A. Search Strategy**

*Global search (all systematic reviews in the COST Action serie)*

**Pubmed:**

MH AND Teaching OR training OR treatment OR "clinical trial" OR intervention OR therapy OR rehabilitation OR remediation OR “special education” OR "dynamic assessment" OR "response to intervention") AND ("language impairment" OR "language delay" OR "language disorder" OR "language disability" OR "language development disorders" OR dysphasi* OR aphasi* OR "developmental communication disorder") AND (child* OR preschool* OR adolescen* OR teenage* OR youth) NOT (adult OR deaf OR autis* OR "hearing impairment" OR "Down syndrome" OR "intellectual disability" OR "traumatic brain injury" OR "acquired brain injury" OR "physical disability" OR "learning disability" OR "severe learning difficulties" OR "severe learning difficulty" OR disease) AND (("2006/01/01"[PDat] : "2015/12/31"[PDat]))

**Web of Science:**

TOPIC: ((teaching OR training OR treatment OR "clinical trial" OR intervention OR therapy OR rehabilitation OR remediation OR "special education" OR "dynamic assessment" OR "response to intervention") AND (language AND (impairment OR delay OR disorder OR disability) OR "language development disorder" OR dysphasi* OR aphasi* OR "developmental communication disorder") AND (child* OR preschool* OR adolescen* OR teenage* or youth) NOT (adult OR deaf OR autis* OR "hearing impairment" OR "Down syndrome" OR "intellectual disability" OR "traumatic brain injury" OR "acquired brain injury" OR "physical disability" OR "learning disability" OR "severe learning difficulties" OR disease)) 2006-2015 excluding Chemical abstracts

**ERIC:**

((teaching OR training OR treatment OR "clinical trial" OR intervention OR therapy OR rehabilitation OR remediation OR "special education" OR "dynamic assessment" OR "response to intervention") AND (language AND (impairment OR delay OR disorder OR disability) OR "language development disorder" OR dysphasi* OR aphasi* OR "developmental communication disorder") AND (child* OR preschool* OR adolescen* OR teenage* or youth) NOT (adult OR deaf OR autis* OR "hearing impairment" OR "Down syndrome" OR "intellectual disability" OR "traumatic brain injury" OR "acquired brain injury" OR "physical disability" OR "learning disability" OR "severe learning difficulties" OR disease))

**PsychInfo:**

((teaching OR training OR treatment OR "clinical trial" OR intervention OR therapy OR rehabilitation OR remediation OR "special education" OR "dynamic assessment" OR "response to intervention") AND (language AND (impairment OR delay OR disorder OR disability) OR "language development disorder" OR dysphasi* OR aphasi* OR "developmental communication disorder") AND (child* OR preschool* OR adolescen* OR teenage* or youth) NOT (adult OR deaf OR autis* OR "hearing impairment" OR "Down syndrome" OR "intellectual disability" OR "traumatic brain injury" OR "acquired brain injury" OR "physical disability" OR "learning disability" OR "severe learning difficulties" OR disease))

**Scopus:**

Was searched, sub-divided into four strings (as SCOPUS would not accept the full string in one entry field):

( TITLE-ABS-KEY ( teaching  OR  training  OR  treatment  OR  "clinical trial"  OR  intervention  OR  therapy  OR  rehabilitation  OR  remediation  OR  "special education"  OR  "dynamic assessment"  OR  "response to intervention" )  AND  TITLE-ABS-KEY ( "language impairment"  OR  "language delay"  OR  "language disorder"  OR  "language disability"  OR  "language development disorder"  OR  dysphasi*  OR  aphasi*  OR  "developmental communication disorder" )  AND  TITLE-ABS KEY ( child*  OR  preschool*  OR  adolescen*  OR  teenage*  OR  youth )  AND NOT  TITLE-ABS-KEY ( adult  OR  deaf  OR  autis*  OR  "hearing impairment"  OR  "Down syndrome"  OR  "intellectual disability"  OR  "traumatic brain injury"  OR  "acquired brain injury"  OR  "physical disability"  OR  "learning disability"  OR  "severe learning difficulties"  OR  disease ) )  AND  SUBJAREA ( mult  OR  medi  OR  nurs  OR  vete  OR  dent  OR  heal  OR  mult  OR  arts  OR  busi  OR  deci  OR  econ  OR  psyc  OR  soci )  AND  PUBYEAR  >  2005  AND  PUBYEAR  <  2016  AND  ( EXCLUDE ( DOCTYPE ,  "le" ) )

**Language and Linguistic Behaviour Abstracts:**

ti(((teaching OR training OR treatment OR "clinical trial" OR intervention OR therapy OR rehabilitation OR remediation OR "special education" OR "dynamic assessment" OR "response to intervention" OR instruction OR therapeutic* OR remedial) AND (language AND (impairment OR delay* OR disorder OR disability OR patholog* OR dysfunction OR "communication disorders" OR "language development disorder" OR

dysphasi* OR aphasi* OR "developmental communication disorder")) AND

(child* OR preschool* OR adolescen* OR teenage* OR youth OR student*)) NOT (adult OR deaf OR autis* OR "hearing impairment" OR "Down syndrome"

OR "intellectual disability" OR "traumatic brain injury" OR "acquired brain injury" OR "physical disability" OR "learning disability" OR "severe learning difficulties" OR disease)) OR ab(((teaching OR training OR treatment OR "clinical trial" OR intervention OR therapy OR rehabilitation OR remediation OR "special education" OR "dynamic assessment" OR "response to intervention" OR instruction OR therapeutic* OR remedial) AND (language AND (impairment OR delay* OR disorder OR disability OR patholog* OR dysfunction OR "communication disorders" OR "language development disorder" OR dysphasi* OR aphasi* OR "developmental communication disorder")) AND (child* OR preschool* OR

adolescen* OR teenage* OR youth OR student*)) NOT (adult OR deaf OR

autis* OR "hearing impairment" OR "Down syndrome" OR "intellectual disability" OR "traumatic brain injury" OR "acquired brain injury" OR "physical disability" OR "learning disability" OR "severe learning difficulties" OR disease))

*Specific terms for the pragmatic domain of the systematic reviews:*

**pragmatic*** OR **social** (communication OR skill OR interaction) OR **conversation*** OR speech OR **narrativ*** OR figurative language (**idiom** OR metaphor OR simile OR proverb) OR non-literal OR **inferen*** (for inferencing or inferential)

Non-literal was excluded as it did not capture any hits
